# Assessment of pain and associated factors in people living with HIV/AIDS*

**DOI:** 10.1590/1518-8345.2803.3155

**Published:** 2019-07-18

**Authors:** Aliny Cristini Pereira, Fernanda Bradbury, Estefani Serafim Rossetti, Priscilla Hortense

**Affiliations:** 1 Universidade Federal de São Carlos, São Carlos, SP, Brasil; 2 Bolsista da Fundação de Amparo à Pesquisa do Estado de São Paulo (FAPESP), Brasil

**Keywords:** HIV, Acquired Immunodeficiency Syndrome, Pain, Pain Measurement, Quality of Life, Depression, HIV, Síndrome da Imunodeficiência Adquirida, Dor, Medição da Dor, Qualidade de Vida, Depressão, VIH, Síndrome de Inmunodeficiencia Adquirida, Dolor, Dimensión del Dolor, Calidad de Vida, Depresión

## Abstract

**Objective:**

to evaluate pain in people living with human immunodeficiency virus/acquired immunodeficiency syndrome and to relate it to sociodemographic and clinical factors, depressive symptoms and health-related quality of life.

**Method:**

descriptive, analytical, observational, cross-sectional and quantitative study. Three hundred and two (302) people assisted at a specialized care service participated in the study. Instruments were used to evaluate sociodemographic and clinical data, depressive symptoms, and health-related quality of life. Descriptive, bivariate analysis and multiple logistic regression were used.

**Results:**

the incidence of pain of mild intensity was 59.27%, recurrent in the head, with interference in mood, mostly affecting females and individuals with no schooling/low schooling. Women were more likely to have moderate or severe pain. People aged 49 to 59 years had greater pain intensity than people aged 18 to 29 years. The variables depressive symptoms and pain were directly proportional. The higher the health-related quality of life and schooling, the lower was the possibility of presence of pain.

**Conclusion:**

presence of pain is of concern and has association with female sex, lack of schooling/low schooling, worse level of health-related quality of life and presence of depressive symptoms.

## Abstract (pt)

**Objetivo:**

avaliar a dor em pessoas que vivem com o vírus da imunodeficiência humana/síndrome da imunodeficiência adquirida e relacionar com fatores sociodemográficos, clínicos, sintomas depressivos e qualidade de vida relacionada à saúde.

**Método:**

estudo descritivo, analítico, observacional, de corte transversal e de caráter quantitativo. Participaram trezentas e duas (302) pessoas de um serviço de atendimento especializado. Foram utilizados instrumentos para avaliação de dados sociodemográficos, clínicos, sintomas depressivos e qualidade de vida relacionada à saúde. Empregou-se análise descritiva, bivariada e regressão logística múltipla.

**Resultados:**

foram encontrados 59,27% de presença de dor de intensidade leve, recorrente na cabeça, com interferência no humor, a maioria do sexo feminino e em indivíduos sem/baixa escolaridade. As mulheres apresentaram maior probabilidade de ter dores moderadas ou severas. Pessoas de 49 a 59 anos apresentaram maior intensidade de dor do que as de 18 a 29 anos. Sintomas depressivos e dor são variáveis diretamente proporcionais. Quanto maior o nível da qualidade de vida relacionada à saúde e escolaridade, menor a possibilidade da presença de dor.

**Conclusão:**

a presença de dor encontrada é preocupante e possui associação com o sexo feminino, sem/baixa escolaridade, pior nível da qualidade de vida relacionada à saúde e presença de sintomas depressivos.

## Abstract (es)

**Objetivos:**

evaluar el dolor en las personas que viven con el virus de la inmunodeficiencia humana/síndrome de inmunodeficiencia adquirida y relacionarse con factores sociodemográficos, clínicos, síntomas depresivos y calidad de vida relacionada con la salud.

**Método:**

estudio descriptivo, analítico, observacional, de corte transversal y de carácter cuantitativo. Participaron trescientos y dos (302) personas de un servicio de atención especializado. Se utilizaron instrumentos para la evaluación de datos sociodemográficos, clínicos, síntomas depresivos y calidad de vida relacionada con la salud. Se empleó el análisis descriptivo, bivariada y regresión logística múltiple.

**Resultados:**

se encontró un 59,27% de presencia de dolor de intensidad leve, recurrente en la cabeza, con interferencia en el humor, la mayoría del sexo femenino y en individuos sin/baja escolaridad. Las mujeres presentaron una mayor probabilidad de sufrir dolores moderados o severos. Las personas de 49 a 59 años presentaron mayor intensidad de dolor que las de 18 a 29 años. Los síntomas depresivos y dolor son variables directamente proporcionales. Cuanto mayor es el nivel de la calidad de vida relacionada con la salud y la escolaridad, menor es la posibilidad de la presencia de dolor.

**Conclusión:**

la presencia de dolor encontrado es preocupante y tiene asociación con el sexo femenino, sin/baja escolaridad, peor nivel de la calidad de vida relacionada a la salud y la presencia de síntomas depresivos.

## Introduction

The global epidemic of the Human Immunodeficiency Virus (HIV), which is the cause of the Acquired Immunodeficiency Syndrome (AIDS), began in 1981 in the United States. After thirty years of fight against HIV/AIDS in Brazil, which started in 1985, the analysis is that this country has been exemplary in its role of prevention and treatment of this disease with great advances and few setbacks^(^ ^1^ ^)^. However, despite the success of disease prevention and control measures, thousands of new infections occur every year. Hence, HIV/AIDS represents a pandemic, becoming a challenge for various social sectors regarding measures to control vulnerabilities involved in the individual and contextual aspects of exposure to the virus^(^ ^1^ ^–^ ^2^ ^)^.

With therapeutic advances and the introduction of new classes of antiretrovirals in the last decades, the status of this infection passed from fatal disease to chronic condition. However, this evolution in treatment is a challenge for patients and health professionals, who in this new context of chronicity must face HIV infection, not as a death sentence, but as a potential hindrance in the life of People that Live with HIV/AIDS (PLWHA)^(^ ^3^ ^)^.

PLWHA present several persistent symptoms that develop during the course of the disease, associated with changes in the immune system and the side effects of antiretroviral therapy (ART). Even when side effects of ART decrease, the prevalence and number of symptoms are still high, regardless of HIV viral load and CD4+ T lymphocyte cell count. The main ones are: fatigue; absence of sleep; muscular pain and pain in the joints and indisposition to work^(^ ^4^ ^–^ ^5^ ^)^.

An outpatient survey in the UK with 859 PLWHA resulted in a prevalence of pain of 62.8%, with respect to pain felt in the last month, of which 63% reported feeling pain on the day of the interview. Among the interviewees, 20% were taking analgesics daily. The study showed that there are many challenges to control pain related to AIDS, including polypharmacy, increased sensitivity to side effects of the drugs, psychological comorbidity and drug interactions^(^ ^4^ ^)^.

A systematic review pointed to several studies reporting the prevalence of pain in PLWHA, and showed that pain is a significant problem but which remains poorly treated. Underdiagnosis and under-treatment of pain in PLWHA are alarming, and few studies have given attention to this topic, and to the records of its occurrence. These data become worrying when it is observed that pain can affect the mood of PLWHA, which can result in depressive symptoms (DS) and negatively impact on life, interfering in the level of Health-related Quality of Life (HRQoL)^(^ ^6^ ^–^ ^8^ ^).^

The understanding of DS and HRQoL in association with pain in PLWHA is fundamental, considering the chronic evolution of the infection, the possibility of greater survival and conviviality with a stigmatizing and transmissible disease, which is incurable to date, with numerous biopsychosocial consequences. Furthermore, this understanding can help health professionals develop pain management strategies through the creation of care protocols^(^ ^9^ ^–^ ^10^ ^)^. A nurse is at the forefront of outpatient care for PLWHA, he play an important role in treatment adherence, controlling pain and other signs and symptoms, enabling better quality of life and satisfaction with treatment.

The purpose of this article is to draw the attention of nurses to the problem of pain and other symptoms in PLWHA and what they can cause in their lives. The nurse’ work in the evaluation of these variables and in the planning of control measures is extremely important for the success of the treatment.

Thus, the present study aims to evaluate pain in people living with human immunodeficiency virus/acquired immunodeficiency syndrome and to relate it to sociodemographic and clinical factors, depressive symptoms and health-related quality of life.

## Method

This is a descriptive, analytical, observational, cross-sectional and quantitative study developed at a Health Service in the city of São Carlos - São Paulo.

To define the sample size, a stratified sampling was performed by sex, starting from the number of PLWHA registered in the registry of the Specialized Care Service (SCS) who were within the age range to be surveyed: 18 and 59 years. There were 1431 individuals in this age group registered in the year 2016. First, the formula for finite populations was used to calculate the sample size. Then, through the national statistics of HIV/AIDS prevalence, the distribution of cases reported in the National Disease Notification System (SINAN) and registered in the System of Control of Laboratory Tests (SISCEL)/System of Logistic Control of Medicines (SICLOM), according to sex and age group between 1980 and 2015. The sum of the cases notified per year of diagnosis made it possible to estimate the distribution of cases by age group and sex. A total of 302 PLWHA, among 197 males and 105 females, were needed to consider the sample as significant.

The inclusion criteria were: to be adult with aged between 18 and 59 years, of both sexes, to have HIV or AIDS, to be assisted at the research site and to be under ART for at least six months.

In this study, it was decided to study only adult individuals because children, adolescents and elderly people are populations that have specific characteristics that deserve to be analyzed in their particularities, which would not be the objective of the present study.

The time of six months under ART was adopted to avoid conflict of interpretation with the variable pain and the beginning of the use of this therapy, because several adverse reactions occur in the first six months of treatment^(^ ^11^ ^)^.

The exclusion criteria were: participants who did not understand or were not able answer the questions of the instruments, women who were pregnant and co-infected people (presenting more than one viral infection).

The invitation to participate in the research was made at the place of consultations. Patients were approached and invited while they were in the waiting room on days when they had scheduled appointments. If the patient accepted, he was given the possibility to choose the most appropriate moment for the interview, that took place in a reserved room to provide the participant with privacy and comfort. The data collection period was from January to July 2017.

Data were collected using public domain questionnaires. Sociodemographic and clinical data related to pain, depressive symptoms and health quality of life were investigated, including the patient's name, age, sex, color/race, marital status, family income, schooling, occupation, CD4+ T lymphocyte count, clinical stage of infection, type and time of ART.

The Brief Pain Inventory (BPI), a tool that was translated, validated and culturally adapted in Brazil in 2011, was used to investigate the pain. BPI evaluates the presence of pain in the last 24 hours, the site of pain, intensity, treatment for relief, percentage of relief, and interference in daily life in 7 dimensions (general activities, mood, ability to move and to carry out normal work, relationships with others, sleep, and enjoyment of life). To characterize the levels of pain intensity, the following scores were used: from 1 to 4, mild pain; from 5 to 7, moderate pain; and from 8 to 10, severe pain^(^ ^12^ ^)^.

The Patient Health Questionnaire-9 (PHQ-9) was used to evaluate the Depressive Symptoms. This questionnaire has nine questions that assess the presence of each of the symptoms of depression. The instrument had its psychometric properties validated in Brazil for the general population in 2013. This study adopted the cutoff point of ≥ 9 for the presence of DS, as recommended by the author^(^ ^13^ ^)^.

The HIV/AIDS Targeted Quality of Life (HAT-QoL) was built specifically for HIV-infected individuals and is used to assess Health-Related Quality of Life (HRQoL). This instrument was translated and validated in Brazil in 2009. The 34 HAT-QoL items evaluate nine HRQoL domains: general function (six items); satisfaction with life (four items); health concerns (four items); financial concerns (three items); concerns with medication (five items); acceptance of HIV (two items); concerns with confidentiality (five items); trust in professionals: physicians; nurses; or any other healthcare professionals who provide care for the patient and sexual function (two items). In each domain, zero is the lowest score and 100 the best score possible. The higher the score, the lower is the impact of HIV infection on the individuals’ HRQoL. In turn, the lower the score, the more affected is the function, the greater is the concern and the lower is the life satisfaction^(^ ^14^ ^)^.

For data analysis, double typing was used to enter data, which were organized in an Excel spreadsheet and exported to the statistical software R (2016). Initially, the consistency of the collected data was checked through descriptive analysis. The normality among sample means was tested with the Kolmogorov-Smirnov and Shapiro-Wilk tests. After that, a bivariate analysis was made to verify the association of sociodemographic and clinical variables with pain. In the case of the categorical variables, the chi-square or Fisher's tests were used, according to the frequency in the classes of the variables. The non-parametric Mann-Whitney test was used to compare pairs of independent groups (with and without pain) in the case of the continuous variables. The level of significance was 5%. Finally, multiple logistic regression models were adjusted with the objective of analyzing the association of all these variables, concomitantly, with pain. Stepwise selection was used to select the significant variables in the model, with an alpha of 10% for inclusion, and alpha of 5% for retention.

This research was approved by the Research Ethics Committee (REC) of the Federal University of São Carlos (UFSCar) under the CAAE n° 62745716.1.0000.5504. The request for its development at the site of data collection was approved by the city hall of the municipality. All participants signed the Informed Consent Term (ICT).

## Results

The results showed that of the 302 PLWHA studied, 179 (59.27%) reported having presented pain in the last 24 hours. In Table 1, the presence of pain was analyzed according to sociodemographic variables, with sex and schooling being the variables with significant values.

**Table 1:** Frequency values (%), absolute values, p-value* of the sociodemographic variables associated with the pain variable in People Living with HIV/AIDS. São Carlos, SP, Brazil, 2017

Variables	Categories	Pain				Total	p-value*
		No		Yes			
		n	%	n	%		
Sex	Female	31	29.52%	74	70.48%	105	0.0038† ‡
	Male	92	46.70%	105	53.30%	197	
Age group (years)	18-≤29	38	45.78%	45	54.21%	83	0.5007‡
	30-≤39	47	39.83%	71	60.16%	118	
	40-≤49	29	40.27%	43	59.72%	72	
	50-≤59	9	31.03%	20	68.96%	29	
Skin color	White	55	45.83%	65	54.17%	120	0.2954‡
	Brown	48	40.34%	71	59.66%	119	
	Black	14	29.79%	33	70.21%	47	
	Others	6	37.50%	10	62.50%	16	
Marital status	Married	26	45.61%	31	54.39%	57	0.2635§
	Divorced	14	46.67%	16	53.33%	30	
	Single	70	42.17%	96	57.83%	166	
	Widowed	2	25.00%	6	75.00%	8	
	Lives with companion	11	26.83%	30	73.17%	41	
Family income	Less than one minimum wage	22	40.74%	32	59.26%	54	0.1327‡
	One minimum wage	39	33.62%	77	66.38%	116	
	Two minimum wages	30	42.86%	40	57.14%	70	
	More than two minimum wages	32	51.61%	30	48.39%	62	
Schooling	No schooling /low schooling	17	35.42%	31	64.58%	48	0.0001† §
	Complete elementary education	19	52.78%	17	47.22%	36	
	Incomplete high school	11	16.92%	54	83.08%	65	
	Complete high school	45	45.45%	54	54.55%	99	
	Incomplete higher education	16	55.17%	13	44.83%	29	
	Complete higher education/Postgraduation	15	60.00%	10	40.00%	25	
Occupation	Recipient of INSS benefit¶ due to HIV**	7	36.84%	12	63.16%	19	0.5215§
	Retired	2	22.22%	7	77.78%	9	
	Unemployed	36	46.15%	42	53.85%	78	
	Employed	78	39.80%	118	60.20%	196	

* p-value = Probability of significance;

† p-value < 0.05;

‡ Chi-square test;

§ Fisher test;

|| 1 minimum wage, Brazil, year 2017 - R$ 937.00;

¶ INSS - National Social Security Institute;

** HIV = Human Immunodeficiency Virus

In Table 2, the presence of pain was analyzed according to the clinical aspects of the disease, and no significant association was found between the clinical variables and the presence of pain.

**Table 2:** Frequency values (%), absolute values, p-value of the clinical variables associated with the pain variable in People Living with HIV/AIDS. São Carlos, SP, Brazil, 2017

Variables	Categories	Pain				Total	p-value*
		No		Yes			
		n	%	n	%		
Lymphocytes CD4+ T	<200 cells/mm³	7	33.33%	14	66.67%	21	0.6139†
	201-350 cells/mm³	13	44.83%	16	55.17%	29	
	351-500 cells/mm³	21	35.00%	39	65.00%	60	
	>500 cells/mm³	82	42.71%	110	57.29%	192	
Clinical stage	2^nd^ phase - asymptomatic	116	41.88%	161	58.12%	277	0.1762†
	4^th^ phase – aids	7	28.00%	18	72.00%	25	
ART§	1^st^ treatment line	81	42.86%	108	57.14%	189	0.7461‡
	2^nd^ treatment line	35	36.46%	61	63.54%	96	
	No treatment	2	33.33%	4	66.67%	6	
	Special situations	5	45.45%	6	54.55%	11	

* p-value = Probability of significance;

† Chi-square test;

‡ Fisher test;

§ ART = Antiretroviral Therapy

The association of the variables CD4 T lymphocytes and pain returned a high p-value, given the level of significance of 5%. Therefore, we do not have evidence to reject the hypothesis that the association between these variables is null, but two aspects are clear in this analysis: there was no perfect ordering in the prevalence, as the concentration of CD4 T cells increased; and the levels of the variable were also not significant in the multiple regression analysis.

Regarding the association between clinical stage and ART, although the categories present apparently different prevalence, the p-value was not significant and it is not possible to reject the hypothesis of null association. However, the lower number of patients in some groups implies the loss of power of the test. The p-value was also not significant in the multiple regression analysis, in both variables.

In Table 3, data on pain are presented with regard to intensity rating and interference of pain in daily life. In general, high levels of interference of pain in the life of PLWHA were observed; mood was highly affected by pain, followed by daily activities and general activities.

**Table 3:** Frequency values (%), central tendency and variability of levels of intensity and interference of pain in different aspects of the life of people living with HIV/AIDS. São Carlos, SP, Brazil, 2017

		n	%	Mean	SD*	Median	Minimum	Maximum
Intensity of pain								
	Mild	108	59.88%	3.26	1.05	3.50	1.00	4.75
	Moderate	65	36.72%	6.11	0.86	6.00	5.00	7.75
	Severe	6	03.38%	8.00	0.52	8.12	7.00	8.50
Interference of pain								
	General activity	179		6.50	7.57	6.71	0.00	10.00
	Daily Activity	179		7.04	3.25	8.00	0.00	10.00
	Mood	179		7.76	3.13	9.00	0.00	10.00
	Ability to walk	179		5.60	4.01	6.00	0.00	10.00
	Job	179		5.82	4.44	8.00	0.00	10.00
	Relationships with other people	179		5.97	3.89	7.00	0.00	10.00
	Sleep	179		4.69	3.78	4.00	0.00	10.00
	Enjoyment of life	179		5.21	3.45	5.00	0.00	10.00

* SD = Standard deviation

In the association of DS with pain, there was a significant result (p < 0.0001). It was possible to observe in the PLWHA that, through the presence of pain, 71.87% had DS, and through the absence of pain, 28.12% had DS.

Regarding the HRQoL, checked with the HAT-QoL, it was observed that in general function (p < 0.0001), satisfaction with life (p < 0.0001), health concerns (p < 0.0001), financial concerns (p < 0.0001), acceptance of HIV (p = 0.0021), concerns about confidentiality (p = 0.0021) and sexual activity (p = 0.0009) showed significant values in the relation with pain. Thus, they indicate that people who have pain present worse HRQoL in these domains.

Only the domains concerns with medication and trust in physicians did not obtain significant values in their relationship with pain, that is, these HRQoL variables are not influenced by pain.

The multiple logistic regression of pain, sociodemographic and clinical data, DS and HRQoL revealed that many variables were not statistically significant at the 5% level. Thus, in order to keep the significant variables in the model, the stepwise selection process was performed (with alpha of retention in the model of 5%). Thus, the mean HRQoL had a p-value < 0.0001 and Odds Ratio (OR) = 0.96. This means each increasing point in the score decreases the chance of PLWHAs reporting pain by 4%. This data points to a inversely proportional relationship between pain and level of HRQoL in this population. It was also observed that another variable resulted to be significant, i.e. sex (p = 0.03). This led to the conclusion that women are 79% more likely than men to report pain.

In Table 4, another model was adjusted and HRQoL was disregarded, due to its high statistical significance in relation to pain. In this case, the variables sex, schooling and DS remained significant.

**Table 4:** Multiple logistic regression without the mean Health Related Quality of Life and with Stepwise selection. São Carlos, SP, Brazil, 2017

Variable	Category	OR*	OR*(95% CI†)		p-value‡
Sex	Female	1.89	1.12	3.20	0.017§
Schooling	Complete high school	0.65	0.38	1.12	0.922§
	Higher education/postgraduation	0.45	0.23	0.88	0.066§
DS||	Score ≥ 9	2.11	1.27	3.52	0.004§

* OR = Odds ratio;

† 95% CI = 95% confidence interval;

‡ p-value = Probability of significance;

§ Stepwise selection (with alpha of retention in the model of 5%);

|| DS = Depressive symptoms

It was observed that the OR for sex was 1.89, meaning that women tend to report pain 89% more than men. In turn, the schooling of PLWHA was inversely proportional to pain. The OR was 0.65, that is, people with higher educational level were 35% less likely to report pain. Therefore, PLWHA with higher educational level tended to report less pain than those with less education than complete secondary education. Regarding DS, when the score was ≥ 9 in the PHQ-9, which means presence of DS, PLWHA had a 2.11-fold greater chance to report pain than PLWHA scoring < 9.

In Table 5, we present the selection of significant variables to classify pain intensity in two large groups. Thus, group 1 (severe pain and moderate pain) was compared to group 2 (mild and no pain). Such analysis was performed to correlate the studied variables with the pain levels present in the study population. In this way, it is possible to visualize possible significant data that can distinguish the levels of pain and, finally, characterize important specificities in the study population.

**Table 5:** Multiple logistic regression of the significant variables for distinction of pain intensity. São Carlos, SP, Brazil, 2017

Variable	Category	OR*	OR* (95% CI†)		p-value‡
Sex	Female	2.07	1.16	3.71	0.014
Age group	> 29 years and <= 49 years	1.29	0.66	2.55	0.189
	> 49 years	3.85	1.40	10.61	0.008
Family income§	Two minimum wages	0.37	0.17	0.78	0.612
	More than two minimum wages	0.21	0.08	0.57	0.038
PHQ-9||	Score ≥ 9	2.48	1.39	4.43	0.002

* OR = Odds ratio;

† 95% CI = 95% confidence interval;

‡ p-value = Probability of significance;

§ 1 minimum wage, Brazil, year 2017 - R$ 937.00;

|| PHQ-9 = Patient Health Questionnaire-9

The data in the table show that sex was once again present with significant value (p = 0.01). The OR indicates that women were 2.07-fold more likely to report moderate or severe pain than men. The age range of PLWHA from 49 to 59 years old presented an OR of 3.85. This means that people in this age group had complaints of pain ranging from moderate to severe approximately 4-fold more frequently than young people (reference 18 to 29 years).

It was possible to observe that as the income increased, there was a decrease in the complaint of moderate/severe pain and that the group of patients with PHQ-9 score ≥ 9 were 2.48-fold more likely to present moderate and severe pain.

## Discussion

Regarding the presence of pain, it was possible to observe that 59.27% of the PLWHA had had pain in the last 24 hours. It is understood that the presence of pain in almost 60% of the PLWHA is a matter of concern. However, it is necessary to know the prevalence of pain in the general population to affirm that this prevalence in PLWHA is really high.

A systematic review was conducted to investigate the prevalence of pain in PLWHA, which resulted in a variation of 54% to 83% in the studies analyzed^(^ ^6^ ^)^. A study carried out in Uganda, with the objective of analyzing the prevalence of pain in PLWHA, resulted in 68% of people reporting pain at the time of interview^(^ ^7^ ^)^.

However, in contrast with the present study, other results showed values of prevalence of pain lower than those found here, but still worrying. In Thailand, researchers showed that only 22% of the PLWHA studied reported pain in the last 24 hours^(^ ^15^ ^)^. Another study analyzed the presence of pain in the last year in PLWHA in New York and found a prevalence of pain of 40% in this population^(^ ^16^ ^)^.

When the pain was correlated with sociodemographic variables, it was possible to observe a predominance of pain in women (70.48%). This correlation was also evidenced in the literature; some studies found strong associations between the female sex and pain^(^ ^17^ ^–^ ^18^ ^)^.

As for educational level, significant values were observed in its relationship with pain. PLWHA with incomplete high school presented higher prevalence of pain (83.08%), followed by people with no schooling/low schooling (64.58%). Two other studies^(^ ^7^ ^,^ ^10^ ^)^ showed a higher prevalence of pain in PLWHA who only had elementary education, followed by PLWHA who had only high school.

Pain resulted to be significantly related (p = 0.0448) to time of ART in the PLWHA; people who felt pain had been under ART for a longer time (5.83 years or ± 60 months) than people who did not feel pain.

The relationship of time under ART with pain has already been reported in the literature. The continued use of ART may cause neurological adverse effects, such as peripheral neuropathic pain. In this context, neuropathy still stands out due to the lack of knowledge about the pain associated with the type of drug or therapeutic scheme used by the patient. Even with the evolution of effectiveness and quality of ART, pain in PLWHA is still common. Thus, it is important to recognize that both the viral replication mechanisms and the continuous use of ART in association with adverse events contribute to the clinical manifestation of pain in this population^(^ ^19^ ^–^ ^20^ ^)^.

Regarding pain levels, the present study showed a more frequent report of mild pain (59.88%), followed by moderate pain (36.72%), and severe pain (3.38%). A study conducted in the state of Rio Grande do Norte, Brazil, resulted in 47.5% of PLWHA showing no pain/mild pain; 24.1%, moderate pain and 28.4%, severe pain^(^ ^18^ ^)^. Pain, in general, is recognized as having a negative impact on the ability to perform daily functions of PLWHA^(^ ^6^ ^)^.

On the interference of pain in the daily life of PLWHA, in the present study, it was observed that the activities that suffered the most intense interference were mood, followed by daily activity and, finally, general activity. The literature shows high interference of pain in daily life, especially in sleep aspects, life enjoyment, and work of PLWHA^(^ ^15^ ^,^ ^21^ ^–^ ^22^ ^)^.

Regarding the areas of the body with greater predominance of pain, the head was the most recurrent area in the present study. Studies have shown similar data, resulting in the identification of the head as one of the most prevalent areas of the body affected by pain^(^ ^4^ ^–^ ^7^ ^,^ ^11^ ^,^ ^16^ ^)^.

As for DS and its relationship with pain, significant values were obtained as a result. In this way, people with pain presented DS in greater proportion. A study found that almost half of PLWHA (42.8%) who had pain also had moderate or severe depression. The direction of the relationship between pain and depression can be reciprocal as it is likely that those who experience pain become depressed and those who are depressed may be more likely to report pain. In addition, changes in the central nervous system of PLWHA associated with depression may influence the evolution of the disease, since they contribute to the increase of biological vulnerability^(^ ^22^ ^–^ ^25^ ^)^.

The absence of relationship between HIV/AIDS and DS has also been evidenced in the literature; two studies showed a weak relationship between the variables. A study evidenced that, although some PLWHA with pain had DS (10.3%), there was no significant relationship between these variables. Another study found that among PLWHA, the prevalence of these comorbidities together was as low as 5%. Although not all patients with pain are depressed, both pain and depression may result from manifestations of diseases that disturb other aspects of life^(^ ^10^ ^,^ ^15^ ^)^.

Through the HAT-QoL instrument, it was possible to observe that the domains general function, satisfaction with life, health concerns, financial concerns, acceptance of HIV, concerns about confidentiality, and sexual activity presented significant values and a lower HRQoL than found in other studies, in which the presence of pain was not investigated. The domains that did not present statistically significant values (concern with medication and trust in professionals), the level of HRQoL was lower than in other comparative studies where only HRQoL of PLWHA were analyzed ^(^ ^9^ ^,^ ^26^ ^)^. Thus, the analysis of the nine domains made clearly visible that pain has a negative impact on the HRQoL of PLWHA.

The multiple logistic regressions performed in the present study indicated that the increase of one point in the HAT-QoL decreased the chance of PLWHA to report pain, i.e. the higher the pain the lower the HRQoL. Two studies reported that moderate to severe pain had a significant impact on functional capacity and HRQoL^(^ ^4^ ^,^ ^21^ ^)^. Thus, pain has a clear debilitating effect on the HRQoL of PLWHA^(^ ^7^ ^)^.

It was also possible to see, in this study, that the highest level of pain was prevalent in women, who had a 79% (OR = 1.05 – 3.05) higher chance of reporting pain and 2.07-fold greater chance of reporting moderate or severe pain. When the HRQoL variable was not considered, due to its high statistical significance in relation to pain, women had 89% (OR = 1.12 – 3.20) chance of reporting pain in relation to men.

The present study also had as result that higher level of schooling was associated with lower pain. It was found that people with a higher level of education had a 35% lower chance of reporting pain. The relationship of pain with individuals with no schooling/low schooling has already been evidenced in the literature, both with regard to the presence and intensity of pain; lower levels of schooling were associated with more intense pain^(^ ^15^ ^,^ ^18^ ^)^.

The present study showed that PLWHA who scored ≥ 9 in the PHQ-9, which indicates the presence of DS, had a 2.11-fold greater chance of reporting pain and 2.48-fold greater chance of presenting moderate or severe pain. Some of the identified factors have been associated with the risk of DS in people infected with HIV, including: the perception that HIV affects all aspects of life (perception of consequences imposed by the disease) and emotional problems associated with the seropositive diagnosis (stress).

Efforts to diagnose and treat an episode of DS associated with HIV can prevent pain and future co-morbidities related to it, and have a positive impact on HRQoL. Therefore, it is important that health professionals know that they will often have to deal with the somatization of emotional complaints and illnesses, in order to promote the mental health of PLWHA. Thus, the nurse play an important role in this situation, helping the health team in the prevention, diagnosis and treatment of DS among PLWHA, as well as in the comprehensive care for these individuals^(^ ^8^ ^,^ ^10^ ^,^ ^27^ ^–^ ^28^ ^)^.

It was observed that PLWHA in the age group of 49 to 59 years presented approximately 4-fold greater chance of complaining of pain ranging from moderate to severe than people in the age group of 18 to 29 years. This result was also present in other studies^(^ ^7^ ^,^ ^18^ ^,^ ^29^ ^)^. One of these studies carried out with PLWHA in outpatient care showed that patients over 36 years of age presented more pain than these with lower ages, with mild pain as the more common, followed by severe and finally moderate pain. In a logistic regression, it was found that people older than 36 years were 1.02-fold (p < 0.310) more likely to develop moderate pain than mild pain/no pain, and 0.99-fold (p < 0.689) more likely to develop severe pain than mild/no pain^(^ ^7^ ^)^.

As for income, as this factor increased, there was a decrease in the claim of moderate and severe pain. A study listed several risk factors impacting pain, and low income was one of them. Thus, studies show higher pain levels in countries where the population has low and average economic income^(^ ^15^ ^,^ ^30^ ^)^.

As a limitation of the study, the analysis related to the presence of pain was restricted to the last 24 hours because of the instrument used for pain evaluation. However, this restriction prevents us from knowing the actual presence of pain. This may have eliminated the possibility of identifying a greater number of people with pain, that is, people who did not present pain in the last 24 hours but presented it frequently in the last week or month, so that they did not have their characteristics evaluated.

## Conclusion

It is noteworthy that the present study presents data about a poorly investigated variable in the country, pain in PLWHA.

The results showed a very worrying prevalence of pain in PLWHA in adulthood, with highlight for a greater association with female sex, low level of schooling, worse level of HRQoL, and presence of DS. These results allow the discussion between researchers and nurses with the intention of designing measures to objectify the management of this symptom.

Nursing professional has a fundamental role in the health team regarding the diagnosis, prevention and management of pain in PLWHA. Thus, in order to enable good HRQoL, nursing actions aimed at the diagnosis, prevention and management of pain in people living with a chronic disease, in which innumerable other symptoms may occur, are extremely important.

Further studies can be developed, for example, with longitudinal monitoring of this sample to understand how these variables behave over time. In addition, studies could also focus on knowing the causes of pain and testing interventions that may minimize it.
